# Contexts Paired with Junk Food Impair Goal-Directed Behavior in Rats: Implications for Decision Making in Obesogenic Environments

**DOI:** 10.3389/fnbeh.2016.00216

**Published:** 2016-11-08

**Authors:** Michael D. Kendig, Ambrose M. K. Cheung, Joel S. Raymond, Laura H. Corbit

**Affiliations:** School of Psychology, The University of SydneySydney, NSW, Australia

**Keywords:** instrumental conditioning, Pavlovian conditioning, stimulus, habit, junk food, context, rat

## Abstract

The high prevalence of obesity and related metabolic diseases calls for greater understanding of the factors that drive excess energy intake. Calorie-dense palatable foods are readily available and often are paired with highly salient environmental cues. These cues can trigger food-seeking and consumption in the absence of hunger. Here we examined the effects of palatable food-paired environmental cues on control of instrumental food-seeking behavior. In Experiment 1, adult male rats received exposures to one context containing three “junk” foods (JFs context) and another containing chow (Chow context). Next, rats were food-deprived and trained to perform instrumental responses (lever-press) for two novel food rewards in a third, distinct context. Contextual influences on flexible control of food-seeking behavior were then assessed by outcome devaluation tests held in the JF, chow and training contexts. Devaluation was achieved using specific satiety and test order was counterbalanced. Rats exhibited goal-directed control over behavior when tested in the training and chow-paired contexts. Notably, performance was habitual (insensitive to devaluation) when tested in the JF context. In Experiment 2 we tested whether the impairment found in the JF context could be ameliorated by the presentation of a discrete auditory cue paired with the chow context, relative to a second cue paired with the JF context. Consistent with the results of Experiment 1, the devaluation effect was not significant when rats were tested in the JF context with the JF cue. However, presenting the chow cue increased the impact of the devaluation treatment leading to a robust devaluation effect. Further tests confirmed that performance in the chow context was goal-directed and that sensory-specific satiety in the JF context was intact. These results show that environments paired with palatable foods can impair goal-directed control over food-seeking behavior, but that this deficit was improved by a cue paired with chow. This has promising implications for assisting individuals in controlling their eating behavior in environments designed to dysregulate it.

## Introduction

Obesity is now widespread across the developed and developing world, with the number of obese individuals recently estimated to exceed that of underweight people worldwide (World Health Organisation, 2016). A key driver of excess energy intake and long-term weight gain is the abundance of highly palatable and energy-dense foods. These products are typically advertised with highly salient cues that are ubiquitous in day-to-day life and which are explicitly designed to influence consumption. For example, one study found that children ate significantly more after viewing advertisements for food than for non-food products, regardless of body weight, and that the amount eaten was positively correlated with how many adverts were recognized (Halford et al., 2004).

A substantial proportion of eating now occurs outside of the home, and these meals are associated with greater energy intake and lower micronutrient content (Stroebele and De Castro, 2004; Lachat et al., 2012). These external environments are riddled with stimuli designed to promote food purchase and consumption. While attending to food cues was highly adaptive in earlier periods of human history, relying too heavily on external cues may undermine body weight regulation in modern environments (Berthoud, 2007, 2012). Indeed, there is ample evidence for stimulatory effects of food cues on consumption in the short term. Animal models of *cue-potentiated feeding* show that cues paired with the delivery of food to hungry rats elicit consumption of this food when rats are no longer food-deprived (Weingarten, 1983; Petrovich, 2013), with similar effects found in people (e.g., Cornell et al., 1989). However, long-term effects of food-cue exposure on weight gain have not been established, in part due to the difficulty of testing this hypothesis. For example, in animal models the effects of food cues are sometimes tested within-subjects (Boggiano et al., 2009) and animals are commonly food deprived to encourage learning of the cue-food association, constraining body weight change (but see Reppucci and Petrovich, 2012).

Of course, food cues may affect eating behavior in ways other than prompting immediate consumption. Food is not always readily available in the presence of food cues; for example, when driving past a fast-food sign or walking through a shopping center food court. In these instances, cues may influence consumption via a series of cognitive processes involving where, what and how much food to procure. How food cues affect the decision-making processes that precede actual consumption is relatively less studied and was the focus of the present experiments. To explore this, we applied a framework based on principles of instrumental learning that distinguishes between behavior that is volitional (i.e., goal-directed) and that which is habitual (Dickinson, 1985). Performance of a goal-directed behavior, such as pressing a lever for food, relies on the contingency between the lever press (action) and food reward (outcome) and the fact that the food reward is currently valued. Therefore, manipulating the value of the reward should produce corresponding changes in performance of the action if the behavior is goal-directed, and no change or a reduced change if the behavior is under habitual control (Dickinson and Balleine, 1994). The outcome devaluation paradigm is a behavioral assay used to determine whether an action is under goal-directed or habitual control. The value of a reward is manipulated either by specific satiety or by inducing sickness (via lithium chloride) and performance of the action that earns the devalued outcome is compared either with conditions where the same outcome is valued or with a second action earning a different outcome for which value is intact (Adams and Dickinson, 1981; Balleine and Dickinson, 1998). Goal-directed behaviors are sensitive to changes in outcome value and, therefore, manifest as a selective reduction of the action earning the devalued outcome. By contrast, behaviors under habitual control are insensitive to changes in outcome value and are evident in responding that is not selectively sensitive to manipulation of the outcome of responding.

Recent studies have shown that habitual control over behavior can be accelerated by chronic access to diets high in sugar and/or fat in rats (Kendig et al., 2013; Furlong et al., 2014) and that higher BMI was associated with reduced sensitivity to devaluation in people (Horstmann et al., 2015). Here we focused not on lasting changes produced by long-term diet but on whether contexts paired with highly palatable foods could alter sensitivity to devaluation. The general experimental procedure was modeled on that used in two studies demonstrating that contexts paired with drugs of abuse promoted habitual control over behavior. In the first, rats were injected with ethanol and placed in one distinct context and injected with saline then placed in another context, prior to instrumental training conducted in a third environment. Devaluation tests revealed that responding was insensitive to devaluation when rats were tested in the alcohol-paired context but goal-directed in the saline context (Ostlund et al., 2010). The second study used a similar procedure to demonstrate habitual control over behavior produced by contexts paired with methamphetamine (Furlong et al., 2015). Importantly, instrumental performance was reinforced with food rather than drug rewards and the animals were drug-free at test, indicating that the contexts, rather than acute intoxication, influenced the decision-making processes that promoted habitual responding. We adopted a similar experimental procedure to Ostlund et al. (2010) and Furlong et al. (2015) to assess whether junk food (JF)-paired contexts would disrupt sensitivity to outcome devaluation.

The two experiments reported here each began with Pavlovian context conditioning in which non-deprived rats received repeated exposures to one context paired with standard lab chow and another paired with highly palatable JFs. Rats were then food-deprived for instrumental training in a third context where two lever-press responses for two novel food rewards were trained. Sensitivity to outcome devaluation was then examined in the JF, chow and training contexts. Experiment 1 found that the JF context promoted habitual control over behavior. Experiment 2 attempted to reverse this effect by exploring whether the presentation of a discrete cue paired with chow and satiety would restore goal-directed control over behavior in the JF context.

## Experiment 1

### Materials and Methods

#### Subjects

All experimental procedures were carried out in accordance with the recommendations of the Australian code for the care and use of animals for scientific purposes 8th edition (2013), and were approved by the Animal Ethics Committee at the University of Sydney. Twenty-eight adult male hooded Wistar rats were used. These animals were tested in two replications (*n* = 16 and *n* = 12) that underwent identical experimental procedures. Rats were sourced from the University of Adelaide, were experimentally naïve, and were group-housed (*n* = 4/cage) in temperature- and humidity-controlled ventilated cages in a colony room maintained on a 12:12 light:dark cycle (lights on 7 am–7 pm). Testing was conducted between 2–5 pm each day. Chow and water were available *ad libitum* during context conditioning, but food access was restricted during instrumental training (see below). Rats were handled regularly prior to the beginning of the experiment.

#### Apparatus

All behavioral procedures were conducted in operant chambers (Med-Associates, St. Alban, VT, USA) contained within light- and sound-attenuating shells. The top and side walls of these chambers were Plexiglas and the floor consisted of steel bars. A recessed magazine was centered on one wall of the chamber between two retractable levers. Illumination was provided by a houselight centered at the top of the wall opposite the levers. For context conditioning, visual, tactile and olfactory cues were used to form two distinct contexts that were paired with JFs and chow in a counterbalanced fashion. Thus, one context contained a smooth plastic floor insert, was scented with vanilla essence (10% v/v in water; Queen, Queensland) and had top and side walls decorated with black and white stripes. The second context was scented with peppermint odor (10% v/v in water; Queen, Queensland), had black spots on a white background surrounding the top and side walls, and contained a floor insert covered with rough sandpaper. Odors were pipetted onto folded paper towels that were inserted into the front edge of the bedding tray. Wall decorations were laminated sheets of paper fitted around the exterior of the chamber. Instrumental training was conducted in the same operant chambers with all cues removed to form a “training” context. The houselight was on during all context conditioning and instrumental training sessions. The rewards used in instrumental training were 45 mg pellets (grain-based formula, BioServ, USA) and 20% w/v sucrose solution (~0.1 ml per reward), which are both highly palatable to rats and greatly preferred to chow. Devaluation pre-feeding was conducted in individual acrylic cages with metal bar tops located in a separate room to operant chambers.

#### Procedure

##### Context conditioning

Context conditioning lasted for 14 days and consisted of seven, 1 h exposures each to the Chow and JF contexts in an alternating sequence (chow, JF, chow, JF, etc.). Laboratory chow (Specialty Feeds^®^; 14.23 kJ/g) was provided in the chow context. In the JF context three palatable foods were provided: Oreos (Nabisco, East Hanover, NJ, USA; 20.33 kJ/g), Pringles (Pringles, Battle Creek, MI, USA; 22 kJ/g), and Jelly Snakes (Nestlé, Australia, 14.2 kJ/g). The total weight of food available in JF and Chow sessions was approximately 15 g. Foods were presented in white ceramic dishes centered against the side wall of the chamber. Food was weighed before and after the session to determine intake, which was converted from grams to kJ for analyses and summed for the three foods in the JF context.

##### Instrumental training

Immediately after day 14 of context conditioning, home-cage chow was removed and a restricted feeding schedule introduced wherein rats were fed 14–15 g of chow per rat each day. Instrumental training began 2 days after the last day of context conditioning with a magazine training session where 20 pellets and 20 sucrose rewards were delivered to the magazine on independent random-time 60 s schedules. The left and right levers were then assigned to earn these rewards in a counterbalanced fashion. For the first 6 days of instrumental training, left and right levers were trained in separate sessions that ended either after 30 rewards were earned or 45 min elapsed. The sessions were separated by a minimum of an hour and whether the pellet or sucrose outcome was trained first was alternated each day. For days 1 and 2 of training each lever press was rewarded (i.e., continuous reinforcement). Thereafter, the reinforcement schedule was increased to random-ratio (RR) 5 on days 3 and 4 and RR10 on days 5–7. On day 7 the two levers were trained in the same session. In this session the left lever was inserted until five rewards were earned and then retracted. After 10 s, the right lever was inserted until five rewards of the other outcome were earned. This sequence repeated until 30 rewards of each outcome were earned, or until 60 min had elapsed. This two-outcome procedure was used for all subsequent re-training days between tests. This procedure is similar to that used by Ostlund et al. (2010) but with a shorter delay between levers.

##### Devaluation tests

Devaluation tests were held in the JF, Chow and Training contexts. The order of these three tests was counterbalanced and test days were separated by a single day of re-training using the two-outcome procedure described for training day 7 above. Devaluation was achieved by specific satiety: rats were placed in individual feeding cages and allowed to consume pellets or sucrose solution *ad libitum* for 1 h. Approximately 15 g pellets or 30 g sucrose solution were provided during pre-feeding; rats never consumed more than these amounts. Rats were familiarized to pre-feeding cages on two occasions for 20-min during instrumental training (after daily sessions). The devalued outcome was held constant across tests and counterbalanced, such that pellets were devalued for half of the rats and sucrose solution was devalued for the other half. Immediately after devaluation treatment rats were transferred to the context (JF, Chow or Training) for a 15-min test. Levers were not inserted for the first 10 min of this test to promote attention toward the contexts. After 10 min, both levers were inserted simultaneously for a 5-min test. Presses were recorded but not reinforced.

##### Data analysis

Consumption of chow and JF in context conditioning sessions (kJ/rat) was analyzed using a (2) × (7) within-subjects ANOVA. The dependent measure during instrumental training was the response rate (lever presses/minute) averaged across pellet and sucrose levers. Response rates across days were analyzed in a within-subjects ANOVA. Responding on devalued and non-devalued levers in the three context tests was compared using a within-subjects (2) × (3) ANOVA. Preliminary analyses included devalued outcome (sucrose or pellets) as an additional between-subjects factor but, as it did not interact with the context (Experiment 1) or cue (Experiment 2) effects of interest, we collapsed across this variable for subsequent analyses. Significant interaction effects were followed by tests of simple effects, results for which *p* < 0.05 were considered statistically significant.

### Results

#### Context Conditioning

**Figure 1:**
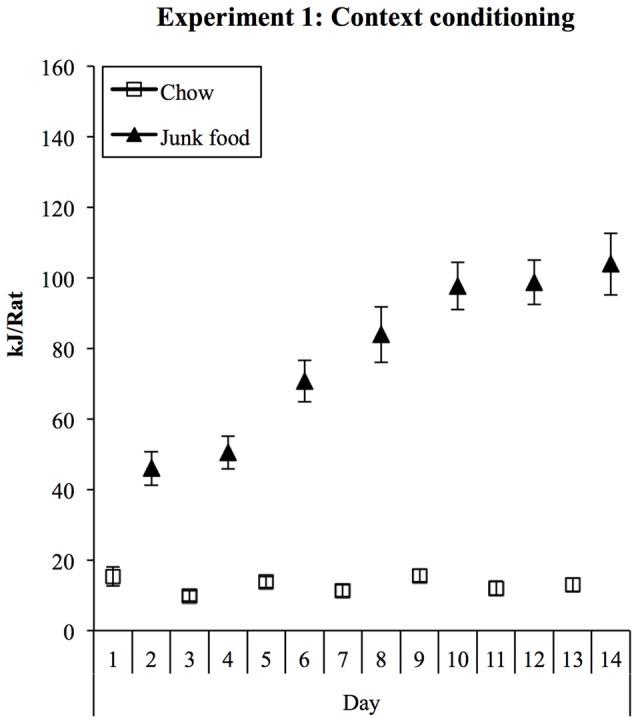
**Experiment 1 context conditioning.** In 1-h daily sessions, rats were exposed to a context containing three “junk” foods (JFs) or to another context containing chow (Chow). Consumption in the JF context was substantially greater from the first session onwards and increased steadily during training, while consumption in the chow context remained low.

Consumption during training is shown in Figure 1. Rats rapidly increased their consumption of JF in the JF context but ate minimal chow in the Chow context. This was supported statistically by a significant effect of session (linear trend: *F*_(1,27)_ = 84.92, *p* < 0.001) and a significant context × session interaction (*F*_(1,27)_ = 79.88, *p* < 0.001) in a (2) × (7) ANOVA. Averaged over sessions, rats ate significantly more in the JF than Chow context (context main effect: *F*_(1,27)_ = 149.30, *p* < 0.001). Despite being non-deprived during this phase, by the end of context training, rats were consuming around eight times more energy in the JF context than in the Chow context.

#### Instrumental Training

**Figure 2:**
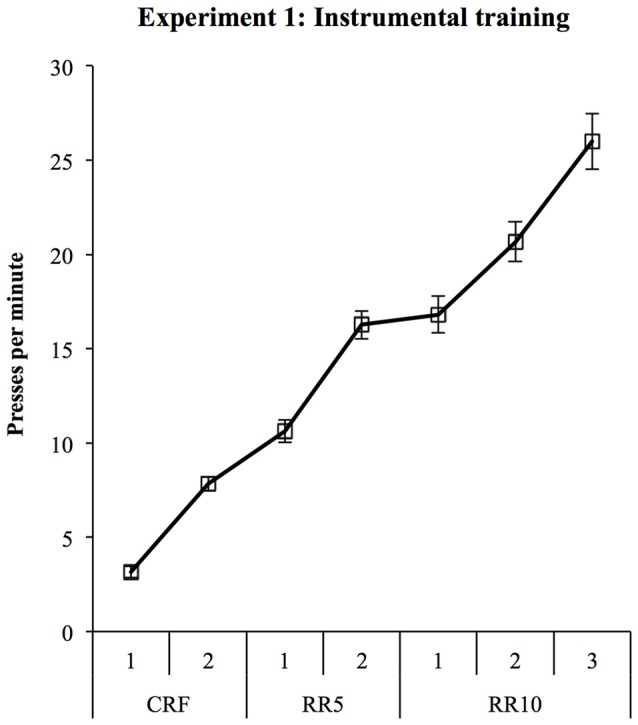
**Experiment 1 instrumental training.** Rats were trained to make two lever presses for pellets and 20% sucrose solution.

All rats learned both instrumental responses. Response rates are shown in Figure 2 and significantly increased during training (linear trend: *F*_(1,27)_ = 301.56, *p* < 0.001). Two rats showed an extreme response bias by responding four times more on the pellet lever than the sucrose lever across training. Since this bias would likely obscure the devaluation effect, these rats were not included in test analyses.

#### Devaluation Tests

##### Pre-feeding

Consumption during pre-feeding did not change significantly over the three test days (*F*_(2,48)_ = 1.53, *p* = 0.227). On average, rats pre-fed with pellets consumed 8.58 ± 0.29 g, while rats pre-fed with sucrose consumed 16.18 ± 0.54 g. However, when expressed as reward equivalents (1 pellet reward = 45 mg and 1 sucrose reward = 0.1 g), consumption was greater in pellet-fed rats (190.6 ± 0.6) than sucrose-fed rats (161.9 ± 5.4). In both cases, consumption far exceeded what rats earned in instrumental training sessions (30 rewards) and rats had stopped eating by the end of the 1-h period, indicating they were satiated.

##### Test

**Figure 3:**
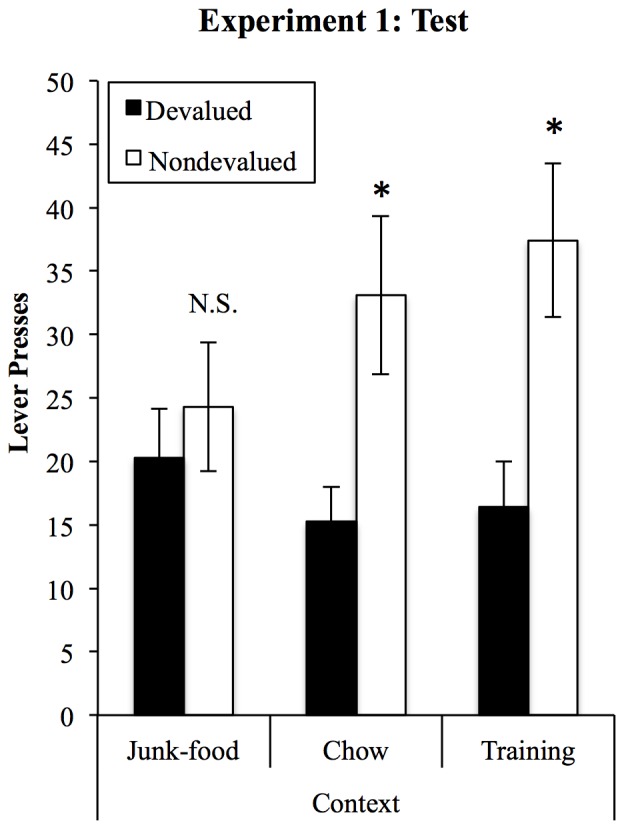
**A JF context impairs sensitivity to outcome devaluation.** Sensitivity to devaluation was tested in the three contexts, within-subjects and in a counterbalanced order. After devaluation of one outcome by specific satiety, rats selectively reduced responding on the lever that had earned that outcome in training, but only in the chow and training contexts. When tested in the JF context, performance was insensitive to devaluation, with no overall difference in responding between contexts. *Indicates *p* < 0.05, N.S., non-significant.

Compiled devaluation test data are displayed in Figure 3 and were analyzed in a (2) × (3) ANOVA (devaluation × context). This analysis found a significant devaluation effect (*F*_(1,25)_ = 5.47, *p* = 0.028) that, critically, interacted with the context in which rats were tested (*F*_(2,50)_ = 3.65, *p* = 0.033). There were no differences in overall responding between contexts (*F* < 1). Sensitivity to devaluation in each context was then assessed using tests of simple effects. These found that rats showed significant devaluation effects when tested in the Training (*F*_(1,25)_ = 7.85, *p* = 0.01) and Chow contexts (*F*_(1,25)_ = 6.27, *p* = 0.019) but that responding on the devalued and non-devalued levers did not differ in the JF context (*F*_(1,25)_ = 0.32, *p* = 0.576).

### Discussion

Experiment 1 trained non-deprived rats to associate one context with highly palatable JFs and another with bland chow. After repeated, alternating exposures to these environments, rats were food-deprived and trained to perform two instrumental responses for distinct food rewards in a third environment. At test we assessed whether the ability to direct food-seeking behavior according to the current value of those foods would be affected by the context in which rats were tested. Rats showed sensitivity to devaluation when tested in a context previously paired with chow or in the environment in which instrumental training occurred. The key finding from this experiment is that these same rats were insensitive to devaluation when tested in the context previously paired with palatable food. Importantly, overall responding did not differ between the three contexts, suggesting that this impairment was not driven by some non-specific effect on overall responding. Rather, rats pressed at a similar rate in this environment but were unable to adjust behavior in accordance with the current value of the outcomes. Therefore, contexts paired with highly palatable JFs undermined goal-directed control over food-seeking behavior.

## Experiment 2

Loss of goal-directed control over food-seeking behaviors could be an obstacle to changing one’s eating behavior. In Experiment 2 we explored whether additional conditioning manipulations could ameliorate this impairment. We modeled our approach on a body of literature studying the effects of discrete stimuli paired with the extinction of previously learned associations, often termed “e-cues”, which are thought to serve as reminders of extinction training and have been shown to promote expression of extinction (Brooks and Bouton, 1993). Under most conditions, extinction of an instrumental response does not erase original learning but rather produces new learning that the response no longer leads to reward. Because responding recovers under a variety of circumstances (Bouton et al., 2012), interventions that protect or strengthen extinction learning are important for reducing these recovery phenomena, particularly in the context of food-related behavior (Bouton, 2011). To this end, Brooks and Bouton (1993) found that a visual cue presented during extinction of a tone-food association (e-cue) attenuated the spontaneous recovery of conditioned responding to the tone when rats were tested 6 days later. Using a similar experimental procedure, Brooks and Bouton (1994) found that presentation of an e-cue prevented ABA renewal, a phenomenon where a response learned in one context (“A”) and extinguished in a second context (“B”) recovers with a return to the first (“A”). A recent study found similar effects of an e-cue on ABA renewal in rats trained to nose-poke for alcoholic beer (Willcocks and McNally, 2014). The typical interpretation of these results is that the presentation of the e-cue facilitates the retrieval of the extinction memory to buffer against returned expression of the original learning (Brooks and Bouton, 1993).

Related to these findings, Ostlund et al. (2010) found that the contextual promotion of habitual responding was reversed by providing response-contingent feedback in the form of outcome delivery. Together, these results suggest that where two conflicting systems compete for behavioral control (original learning vs. extinction, or goal-directed vs. habit systems), stimuli that “remind” the rat of extinction or the devalued state of the outcome can influence behavior to favor the cued learning. Thus, in Experiment 2 rather than attempting to extinguish the JF context, we examined whether a reminder of a relatively unpalatable food; chow, could override the effects of the context previously paired with the palatable JF and promote sensitivity to devaluation. To this end, we presented discrete auditory stimuli in the JF and Chow contexts so that consumption of JF and Chow were paired with a “JF-cue” and a “Chow-cue” in addition to the contexts. We then assessed whether presentation of the Chow-cue would improve sensitivity to devaluation in the JF context relative to when the JF cue was presented in this environment. An additional aim of Experiment 2 was to measure sensitivity to devaluation in terms of consumption as well as instrumental responding. We hypothesized that, just as the e-cue reminds rats of conditions of non-reinforcement (e.g., Willcocks and McNally, 2014), or as outcome delivery reminds animals of changes in outcome value following devaluation (Ostlund et al., 2010), presenting a cue previously paired with chow would remind rats of reduced palatability, and/or satiety, to enhance sensitivity to devaluation in the JF context.

### Materials and Methods

#### Subjects

Twenty adult male Long-Evans rats were used. Animals were bred in-house at the Brain and Mind Centre at the University of Sydney, Australia, and were housed 2–4 per cage in ventilated cages contained in a temperature- and humidity-controlled room. The colony room was maintained on a 12:12 reverse dark:light cycle (lights off 9 am–9 pm). Behavioral testing occurred between 2–6 pm each day. During context conditioning rats had free access to chow and water in home cages. During instrumental training rats were fed approximately 12 g chow daily. Rats were handled regularly in the week prior to the start of the experiment.

#### Design

Context conditioning in Experiment 2 was identical to Experiment 1 except that a discrete auditory cue was also paired with each context. These cues were a white noise and pure tone and were paired with Chow and JF contexts in a counterbalanced fashion. Ten 2-min presentations of these stimuli occurred in every 1-h training session and were separated by a variable ITI (range: 1–4 min). To prevent hearing an inappropriate stimulus from adjacent boxes, rats were run in two groups of 10 rats according to stimulus type. Home-cage chow intake was monitored each day during context conditioning. On the day after the last context conditioning session, rats were pre-exposed in home cages for 2 h to pellets (Bioserv; grain-based formula) and 20% sucrose solution, the outcomes to be used for instrumental training. Food was then removed overnight, and from the following day the restricted feeding scheduled was introduced. Instrumental training was conducted as described for Experiment 1 except that two sessions of the two-outcome procedure were held prior to tests (rather than one).

The first two devaluation tests were conducted in the JF context and compared the effects of the JF and Chow cues (order counterbalanced). Devaluation was achieved by specific satiety as in Experiment 1. For the first 10 min of each test no levers were available and no stimuli were presented. After 10 min both levers were inserted for a 5-min choice extinction test. When levers were inserted, either the Chow- or JF-cue was turned on and played constantly for the remainder of the test. Lever presses were recorded in 1-min bins. On the following day rats received a single session of instrumental re-training using the two-outcome procedure described above. The second devaluation test was identical to the first except that rats tested with the chow cue in Test 1 now received the JF cue, and vice versa. Rats were then given 3 days of re-training prior to a second set of devaluation tests held in the chow context in order to confirm goal-directed responding in this context and test whether the presence of the JF cue was sufficient to impair sensitivity to devaluation. Rats pre-fed with pellets for Tests 1 and 2 were pre-fed with sucrose solution for these tests, and vice versa. The order in which JF- and Chow-paired cues were tested was counterbalanced, and for each rat was the reverse of the order used in tests 1 and 2.

We were also interested to examine whether the habitual performance in the JF-paired context could be explained by impaired sensitivity to sensory specific satiety in this context. Therefore, we examined whether rats would selectively reduce *consumption* of the pre-fed outcome in the JF-paired context. For this test, rats were pre-fed either with pellets (*n* = 10) or sucrose solution (*n* = 9) for 1-h in devaluation pre-feeding cages before a 10-min test of pellet consumption in the JF context with the JF-cue played continuously. Pellets were the test food for all tests due to the logistical difficulty of fixing a bottle of sucrose within operant chambers.

### Results

#### Context Conditioning

**Figure 4:**
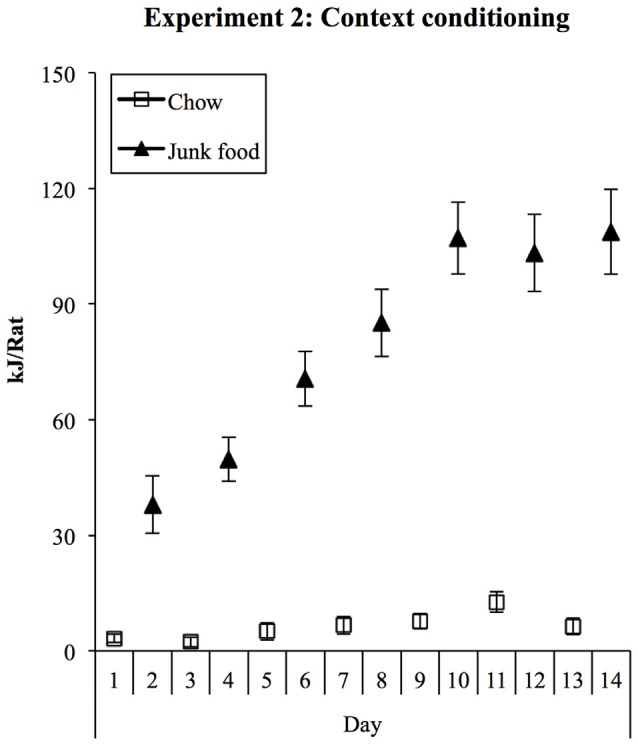
**Experiment 2 context conditioning.** As in Experiment 1, rats rapidly increased their intake of palatable foods in the JF context and ate minimal amounts of chow in the chow context.

Consumption in Chow and JF sessions during training is shown in Figure 4. As in Experiment 1, rats ate substantial amounts of the palatable foods in the JF context but little chow in the Chow context. Consumption of all foods was converted to kilojoules, summed across the three foods in the JF context, and analyzed in a (2) × (7) repeated-measures ANOVA. This analysis showed a significant increase in consumption across sessions (*F*_(1,19)_ = 40.28, *p* < 0.001) and a significant interaction between context and session (*F*_(1,19)_ = 31.633, *p* < 0.001), indicating a greater increase in consumption in the JF- than Chow-paired context. Averaged over sessions, consumption was greater in the JF context (*F*_(1,19)_ = 150.71, *p* < 001).

**Figure 5:**
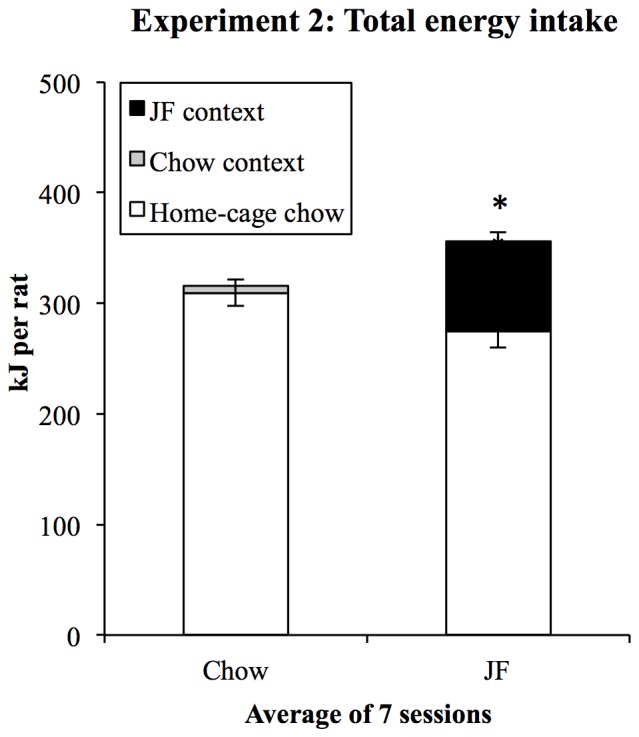
**Total energy intake during context conditioning in Experiment 2.** On average, rats ate more in 24-h periods beginning with a JF context session *p = 0.002. Since rats were group-housed, consumption in daily context sessions was summed for each cage.

Each day, home-cage chow intake was measured when rats were in context conditioning sessions. Total energy intake was then calculated on a per-cage basis by adding home-cage chow intake to the total consumption in the context session by the rats in each cage. Consumption in each day’s training session (kJ/rat) was added to home-cage consumption (kJ/rat) in the following 24-h. This resulted in a measure of 24-h energy intake for each of the six cages on each day of training. Subsequently, we compared total energy intake between chow and JF-training days to assess the extent to which rats compensated for the kJ consumed in JF sessions. Total daily energy intakes were analyzed in a within-subjects (2) × (7) ANOVA, with day type (JF- or Chow-paired day) and “session” as factors. This analysis found a main effect of “day type” (*F*_(1,5)_ = 38.86, *p* = 0.002) indicating that energy intake was higher on days beginning with a JF-session. The difference in average total energy intake indicated by this result is shown in Figure 5. There was no significant linear change in energy intake over days (*F*_(1,5)_ = 6.17, *p* = 0.056).

#### Instrumental Training

**Figure 6:**
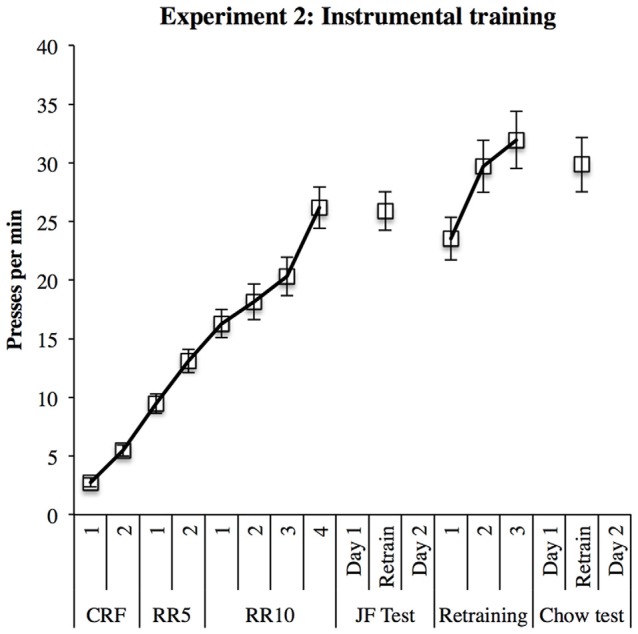
**Instrumental training in Experiment 2.** Responding increased steadily throughout training and remained high during re-training sessions between outcome devaluation tests.

Nineteen rats learned both instrumental responses; the 20th failed to respond for sucrose solution and therefore could not be tested. Average daily responding is displayed in Figure 6. Responding in the first block of training prior to the first test was analyzed in a within-subjects ANOVA. This analysis found a significant linear increase in response rates over sessions (*F*_(1,18)_ = 154.19, *p* < 0.001). These response rates were maintained throughout subsequent re-training sessions.

#### Tests

##### Pre-feeding

Familiarization to the pre-feeding cages was as described for Experiment 1. Rats were pre-fed either with sucrose solution or pellets for both JF context tests, and the other reward (pellets or sucrose) for both Chow context tests. On average, rats consumed 14.13 ± 0.87 g sucrose and 7.81 ± 0.45 g pellets; this was equivalent to 179.32 ± 8.43 pellet rewards and 144.53 ± 8.54 sucrose reward.

##### Effects of the JF- and Chow-cues on sensitivity to devaluation in the JF context

**Figure 7:**
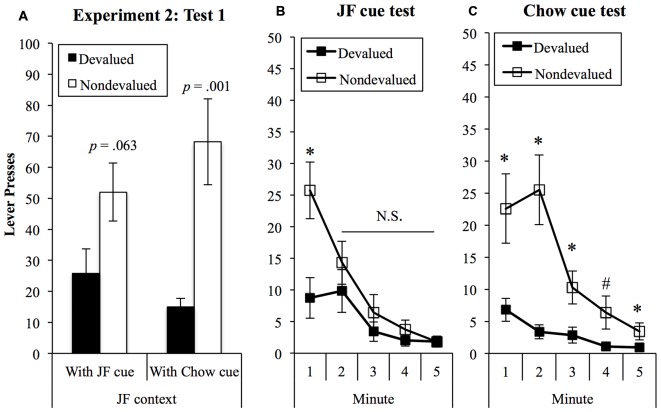
**Devaluation tests in the JF contexts. (A)** Sensitivity to devaluation in the JF context was significantly improved when the chow cue was presented (interaction *p* = 0.038). *p*-values show tests of simple effects in each cue test. Analysis of bin data showed that sensitivity was lost rapidly in the presence of the JF cue **(B)** but remained statistically significant throughout the chow-cue test **(C)**. *p < 0.05; ^#^*p* = 0.057.

Presses on the devalued and non-devalued levers in the chow-cue and JF-cue test are shown in Figure 7A and were analyzed in a (2) × (2) within-subjects ANOVA. This analysis found a significant effect of devaluation (*F*_(1,18)_ = 11.14, *p* = 0.004) and no main effect of cue (*F* < 1). Importantly, there was a significant interaction between devaluation and cue (*F*_(1,18)_ = 4.99, *p* = 0.038), indicating that sensitivity to devaluation treatment varied according to whether the JF- or Chow-paired cue was present during the test. Simple effects analyses were then conducted to explore the nature of the interaction. These analyses found a significant devaluation effect when the Chow-cue was presented in the JF context (*F*_(1,18)_ = 15.54, *p* = 0.001) but not when the JF-cue was presented (*F*_(1,18)_ = 3.93, *p* = 0.063).

To explore the devaluation × cue interaction in greater detail, we examined 1-min bin data for JF-cue and chow-cue tests, shown in Figures 7B,C, respectively. Examining these data suggested that initial sensitivity to devaluation in both tests was rapidly lost in the presence of the JF-cue, but sustained by the chow-cue. To examine this, we added “bin” as a third factor with five levels to a 3-way within-subjects (5) × (2) × (2) ANOVA. This analysis found a significant 3-way interaction between cue, lever and bin (*F*_(4,72)_ = 3.09, *p* = 0.021), indicating that the difference between responding on devalued and non-devalued levers over the five bins varied between JF- and Chow-cue tests. In the JF-cue test, the devaluation effect was significant in the first minute (*F*_(1,18)_ = 6.64, *p* = 0.019) but not in minutes 2, 3, 4, or 5 (largest *F*_(1,18)_ = 1.09). By contrast, during the Chow-cue test the devaluation effect was significant during all five 1-min bins, save for a marginally significant result in minute 4 (minute 1: *F*_(1,18)_ = 6.95, *p* = 0.017; minute 2: *F*_(1,18)_ = 16.02, *p* = 0.001; minute 3: *F*_(1,18)_ = 6.18, *p* = 0.023; minute 4: *F*_(1,18)_ = 4.13, *p* = 0.057; minute 4: *F*_(1,18)_ = 5.95, *p* = 0.025).

##### Sensitivity to devaluation in the Chow context

**Figure 8:**
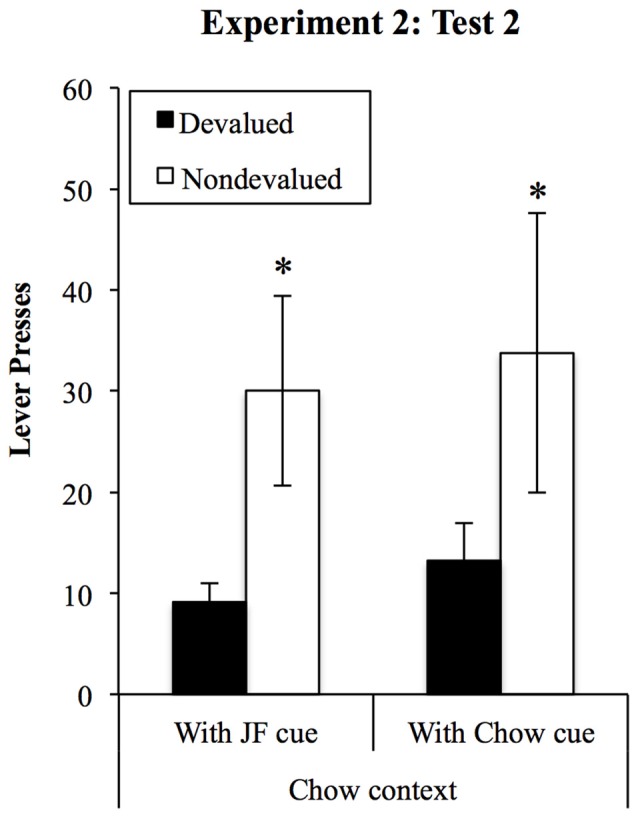
**Devaluation tests in the Chow context.** Performance was sensitive to outcome devaluation and unaffected by which cue was presented when rats were tested in the chow context, despite an overall reduction in responding from the first set of tests. *Indicates *p* = 0.015 for devaluation effect.

Responding on the devalued and non-devalued levers in the chow context tests is shown in Figure 8. The effects of the JF- and Chow-paired cues on performance were assessed using a (2) × (2) within-subjects ANOVA, with cue (JF and Chow) and lever (devalued vs. non-devalued). This analysis found a significant devaluation effect (*F*_(1,18)_ = 7.17, *p* = 0.015) but no effect of cue (*F* < 1) and no interaction between cue and lever (*F* < 1).

##### Sensitivity to devaluation measured with consumption in the JF context

**Figure 9:**
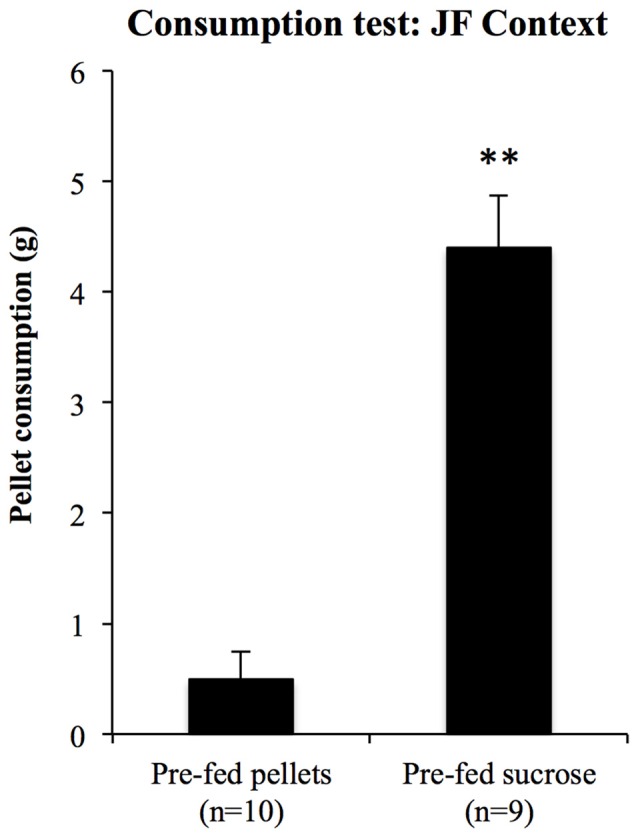
**Sensitivity to outcome devaluation measured by consumption.** To test whether sensory specific satiety was intact in the JF context, pellet consumption in this context was compared between rats pre-fed with pellets and sucrose solution. Those pre-fed with pellets ate significantly less than those pre-fed with sucrose solution, **indicates *p* < 0.001.

For the consumption test in the JF context, 10 rats were pre-fed with pellets and nine were pre-fed with sucrose solution for 20 min prior to a 10-min test of pellet consumption in the JF context. Consumption during test is shown in Figure 9. Pellet consumption in the JF context was significantly reduced in rats pre-fed with pellets relative to those pre-fed with sucrose solution (*F*_(1,17)_ = 57.29, *p* < 0.001); thus, specific satiety itself was intact even when rats were tested in the JF context.

### Discussion

In Experiment 2, we tested whether the effects of the JF context on sensitivity to devaluation would be affected by the presentation of discrete cues paired with JF and Chow. Results indicated that presenting the Chow cue in the JF context improved sensitivity to the devaluation treatment and promoted goal-directed performance across the 5-min of the test. By contrast, when the rats were tested in the JF context with the cue that was present in this context during training, the devaluation effect was not statistically significant. Because numerically the impact of the JF context did not appear as complete as in Experiment 1, analysis of bin data characterized this effect further, showing that the devaluation effect was significant in the first minute of the test but not in minutes 2–5. The initial sensitivity to devaluation may be because the JF context was somewhat degraded at the beginning of the test due to the absence of the auditory cue that was present during training and which was likely to have been a salient element of the context. Thus, in the first 10 min of the test, the absence of the JF-cue rendered the context incomplete. As the onset of the cue “completed” the context, goal-directed behavior was then undermined, but this effect was not apparent until the second minute of the test. Future studies could examine the effects of the auditory cues alone (or other individual elements of the context) to examine their contribution to the observed effect.

Next, we confirmed that sensitivity to devaluation was intact in the Chow context and unaffected by the presentation of the JF- or Chow-cue. These tests were conducted separately and after a period of re-training, because our primary aim was to test a means for restoring goal-directed control in the JF context after observing impaired performance in this environment. A consequence of this approach is that the order of the four devaluation tests was not fully counterbalanced. Not surprisingly, overall responding was lower in the chow context tests (compare Figures 7A, 8) likely due to cumulative extinction of responding resulting from the multiple tests. Importantly, significant devaluation effects were still found in both tests.

Consumption of JFs in the JF context steadily increased over context conditioning sessions such that, by the seventh exposure to this context, rats consumed approximately 30% of their daily calories in a single hour. It is possible, then, that rats associated the JF context not only with palatable tastes, but also with satiety signals and—perhaps—resistance to this satiety. Indeed, studies of cue-potentiated feeding find that contextual food cues can promote consumption even in non-deprived rats that have been pre-fed with the test food (Petrovich et al., 2007). Therefore, we explored whether insensitivity to devaluation in the JF context could be explained by poorer sensory-specific satiety in this environment and to rule out whether altered expression of satiety specifically within the JF context undermined the effectiveness of the devaluation treatment. Results of the consumption test in the JF context showed that this was not the case: rats pre-fed with pellets ate significantly less of that same food than did rats pre-fed with sucrose, indicating that consumption was sensitive to the current value of pellets. The impact of this treatment, however, did not translate into changes in instrumental performance.

## General Discussion

The present experiments sought to further understand how food cues alter food-seeking behavior in ways distinct from consumption. Rats learned to associate one context with the consumption of highly palatable JFs and another with chow, prior to instrumental training conducted in a third environment. We then compared whether these contexts would modulate sensitivity to outcome devaluation. Experiments 1 and 2 found that rats failed to show sensitivity to devaluation in an environment previously paired with consumption of palatable foods. By contrast, rats’ performance was goal-directed when tested in the context previously paired with chow and in the training context. Presentation of a discrete cue previously associated with chow restored sensitivity to devaluation when rats were tested in the JF context. Importantly, these effects of context (Experiment 1) and of the chow-cue (Experiment 2) were not attributable to floor or ceiling effects in responding. Rather, it was the distribution of responding between devalued and non-devalued levers that was impaired by the JF context in Experiment 1. Likewise, presentation of the chow-cue in Experiment 2 significantly improved the ability to direct responding toward the non-devalued outcome.

The current findings are consistent with past studies showing similar impairments in sensitivity to devaluation in contexts paired with ethanol (Ostlund et al., 2010) and methamphetamine (Furlong et al., 2015). While rats ate more JF than chow, and thus may have associated eating freely available food with the JF context which may, in some way, have interfered with having to earn food, as noted above, rats continued to respond in the JF context, they just did so indiscriminately. Furthermore, given the similarity between the current results and those seen in drug-paired contexts, it seems unlikely that the results can be explained by previous consumption. The novel result of the present study is that presentation of a chow-cue significantly improved performance in the JF context.

Although caution should be taken when comparing across experiments, it is worth noting that the reduction in goal-directed control within the JF context appeared more complete in Experiment 1. Responding on devalued and non-devalued levers in the JF context was all but equivalent in Experiment 1, but in the comparable test in Experiment 2 (JF context with JF cue) the devaluation effect approached statistical significance (*p* = 0.063, see Figure 7A). We are confident, however, that this does not reflect inadequate statistical power: all testing was within-subjects, and the above result was generated from the data of 19 animals, which is highly powered to detect devaluation effects. Moreover, the most important result in Experiment 2 was that performance in the JF-context was significantly improved by the presentation of the Chow-cue, as supported by a significant interaction between cue and devaluation. Here it may be useful to consider that, while goal-directed and habit-based control are conceptualized as distinct systems competing for control over behavior (Corbit, 2016), variability within them is still meaningful. Thus, the transition from goal-directed to habitual control over behavior does not occur instantaneously, but instead shifts gradually with extended training (Dickinson et al., 1995) and can be accelerated by exposure to drugs of abuse (e.g., Nelson and Killcross, 2006) or to high-sugar/high-fat diets (Kendig et al., 2013; Furlong et al., 2014). A relevant parallel to consider is that extinction-paired “E-cues” reduce, rather than completely block, the relapse from extinction produced by various manipulations (Brooks and Bouton, 1993, 1994; Willcocks and McNally, 2014). In the present studies, goal-directed behavior was significantly poorer in a context associated with highly palatable food and, in turn, was improved by a discrete cue paired with chow. These incremental changes in sensitivity to devaluation are relevant to food-seeking because the regulation of energy intake is as much a question of what and how much to eat as it is whether to eat or not (Wansink, 2004). Both of the present experiments demonstrated poorer sensitivity to devaluation in the JF context, despite differences in the extent of this impairment, while Experiment 2 demonstrated that the presentation of the chow-cue significantly improved performance.

The use of a “chow cue” in Experiment 2 drew from literature exploring how discrete cues paired with extinction protect against the recovery of the original response that occurs following various manipulations (e.g., renewal, reinstatement etc.; Bouton, 2002). However, an important difference in our approach was that the cue we used to “rescue” performance was not associated with extinction of the JF context but rather had been paired with another distinct environment paired with chow. It is worth noting that consumption of chow during context conditioning was minimal. Therefore, it is difficult to determine the extent to which rats associated the chow cue with chow consumption and the relative value of chow in a non-deprived state, or with an environment in which JFs were unavailable. However, it seems likely that any association formed with chow itself would be with its taste and relative palatability upon sampling, given consumption was appreciable, but low. Therefore, presenting this cue in the JF context may have primed memory of the less-palatable chow or, possibly, of the other elements of the chow context. Importantly, the chow cue was not simply a distraction in the JF context, since overall response rates were unaffected. Instead, instrumental responding was better distributed toward the currently-valued outcome in the presence of the chow cue, indicating some restoration of evaluative processes guiding instrumental performance. By contrast, the high levels of JF consumption in the JF context provided opportunity for the JF cue to become associated with the palatable taste and hedonic properties of the JFs and, potentially, with short-term satiety occurring toward the end of the 1-h conditioning session. Regardless, when this cue was presented in the chow-context (in Test 2) responding was still goal-directed. In summary, our data indicate that the chow cue was effective in disrupting the influence of the JF context to promote goal-directed performance like that seen in the Chow-context, perhaps by retrieving some aspect of that that context, or the chow within it, to improve the efficacy of the devaluation treatment.

The current experiments demonstrated contextual influences on sensitivity to devaluation using a within-subjects design. This is an interesting complement to past research showing that chronic exposure to diets high in sugar, or sugar and fat, promotes habitual performance as assessed by outcome devaluation (Kendig et al., 2013; Furlong et al., 2014). Taken together, these results show that highly palatable foods can impair sensitivity to devaluation both transiently (i.e., the current results) and over the longer term. It is interesting to speculate that in people, repeated exposures to palatable food-paired environments might come to disrupt decision-making processes that alter what and how much individuals eat in these environments. In turn, this increases consumption of high-fat, high-sugar foods contained in these environments, predisposing individuals toward a more lasting expression of habitual behavior toward foods. This tentative suggestion bears some resemblance to the “vicious cycle” model of obesity posited by Davidson et al. (2005) which centers on environmental factors that produce and perpetuate hippocampal insult (see also Hargrave et al., 2016). Hippocampal effects would not appear to contribute to the present results, since sensitivity to devaluation is unaffected by lesions of the hippocampus (Corbit and Balleine, 2000) and the shift between goal-directed and habitual performance instead relies on functional changes to corticostriatal circuits (Corbit, 2016).

In summary, the key message from the present experiments is that decision-making processes can be altered by diet and environments associated with consumption of highly palatable foods. Entering an environment where a certain food type is routinely consumed may bias decision-making processes that mediate future food choices. In places where there has been a history of eating so-called JFs—for example, food courts—this conditioning history may predispose people toward poorer food choices and perpetuate consumption of JFs. This might manifest as a decision to buy food despite a recent meal; selecting a less healthy option; or continuing to eat when no longer hungry. Our data also suggest that relatively simple interventions, such as reminders of reduced food value or interrupting the automatic processing of JF cues, might assist individuals in restoring control in environments where control over eating behavior is compromised. Smartphone apps designed to encourage healthy food choices and prevent “binge” episodes are one example, though their efficacy is still unclear, at least in clinical populations (e.g., Fairburn and Rothwell, 2015). Other manipulations of the external environment may also be effective. For example, one study found that college students selected healthier food options when signs throughout a food court highlighted healthy rather than unhealthy foods (e.g., salads vs. burgers; Mollen et al., 2013). A specific hypothesis prompted by the present results is to test whether a chow-paired cue produces similarly beneficial effects in animals showing habit-based performance following chronic diet exposure of the kind described above.

## Author Contributions

MDK conducted experiments with assistance from AMKC and JSR. MDK and LHC drafted the manuscript with assistance from AMKC and JSR. Statistical analyses were performed by MDK. LHC conceived and directed the project. All authors approved the final submission of the manuscript.

## Funding

These experiments were supported by a University of Sydney Bridging Support grant to LHC.

## Conflict of Interest Statement

The authors declare that the research was conducted in the absence of any commercial or financial relationships that could be construed as a potential conflict of interest.

## References

[B1] Adams C. D. Dickinson A. (1981). Instrumental responding following reinforcer devaluation. Q. J. Exp. Psychol. 33, 109–121. 10.1080/14640748108400816

[B2] Balleine B. W. Dickinson A. (1998). The role of incentive learning in instrumental outcome revaluation by sensory-specific satiety. Anim. Learn. Behav. 26, 46–59. 10.3758/bf03199161

[B3] Berthoud H. R. (2007). Interactions between the “cognitive” and “metabolic” brain in the control of food intake. Physiol. Behav. 91, 486–498. 10.1016/j.physbeh.2006.12.016 17307205

[B4] Berthoud H. R. (2012). The neurobiology of food intake in an obesogenic environment. Proc. Nutr. Soc. 71, 478–487. 10.1017/S0029665112000602 22800810 PMC3617987

[B5] Boggiano M. M. Dorsey J. R. Thomas J. M. Murdaugh D. L. (2009). The Pavlovian power of palatable food: lessons for weight-loss adherence from a new rodent model of cue-induced overeating. Int. J. Obes. (Lond) 33, 693–701. 10.1038/ijo.2009.57 19350040 PMC2697275

[B6] Bouton M. E. (2002). Context, ambiguity and unlearning: sources of relapse after behavioral extinction. Biol. Psychiatry 52, 976–986. 10.1016/s0006-3223(02)01546-9 12437938

[B7] Bouton M. E. (2011). Learning and the persistence of appetite: extinction and the motivation to eat and overeat. Physiol. Behav. 103, 51–58. 10.1016/j.physbeh.2010.11.025 21134389

[B8] Bouton M. E. Winterbauer N. E. Todd T. P. (2012). Relapse processes after the extinction of instrumental learning: renewal, resurgence and reacquisition. Behav. Processes 90, 130–141. 10.1016/j.beproc.2012.03.004 22450305 PMC3355659

[B9] Brooks D. C. Bouton M. E. (1993). A retrieval cue for extinction attenuates spontaneous recovery. J. Exp. Psychol. Anim. Behav. Processes 19, 77–89. 10.1037/0097-7403.19.1.77 8418218

[B10] Brooks D. C. Bouton M. E. (1994). A retrieval cue for extinction attenuates response recovery (renewal) caused by a return to the conditioning context. J. Exp. Psychol. Anim. Behav. Processes 20, 366–379. 10.1037/0097-7403.20.4.366 8418218

[B11] Corbit L. H. (2016). Effects of obesogenic diets on learning and habitual responding. Curr. Opin. Behav. Sci. 9, 84–90. 10.1016/j.cobeha.2016.02.010

[B12] Corbit L. H. Balleine B. W. (2000). The role of the hippocampus in instrumental conditioning. J. Neurosci. 20, 4233–4239. 10818159 10.1523/JNEUROSCI.20-11-04233.2000 PMC6772620

[B13] Cornell C. E. Rodin J. Weingarten H. (1989). Stimulus-induced eating when satiated. Physiol. Behav. 45, 695–704. 10.1016/0031-9384(89)90281-3 2780836

[B14] Davidson T. L. Kanoski S. E. Walls E. K. Jarrard L. E. (2005). Memory inhibition and energy regulation. Physiol. Behav. 86, 731–746. 10.1016/j.physbeh.2005.09.004 16263144

[B15] Dickinson A. (1985). Actions and habits: the development of behavioural autonomy. Philos. Trans. R. Soc. Lond. Biol. Sci. 308, 67–78. 10.1098/rstb.1985.0010

[B16] Dickinson A. Balleine B. W. (1994). Motivational control of goal-directed action. Anim. Learn. Behav. 22, 1–18. 10.3758/bf03199951

[B17] Dickinson A. Balleine B. Watt A. Gonzalez F. Boakes R. A. (1995). Motivational control after extended instrumental training. Anim. Learn. Behav. 23, 197–206. 10.3758/bf03199935

[B18] Fairburn C. G. Rothwell E. R. (2015). Apps and eating disorders: a systematic clinical appraisal. Int. J. Eat. Disord. 48, 1038–1046. 10.1002/eat.22398 25728705 PMC4737215

[B19] Furlong T. M. Jayaweera H. K. Balleine B. W. Corbit L. H. (2014). Binge-like consumption of a palatable food accelerates habitual control of behavior and is dependent on activation of the dorsolateral striatum. J. Neurosci. 34, 5012–5022. 10.1523/JNEUROSCI.3707-13.2014 24695718 PMC6802720

[B20] Furlong T. M. Supit A. S. Corbit L. H. Killcross S. Balleine B. W. (2015). Pulling habits out of rats: adenosine 2A receptor antagonism in dorsomedial striatum rescues meth-amphetamine-induced deficits in goal-directed action. Addict. Biol. [Epub ahead of print]. 10.1111/adb.12316 26515740 PMC4851927

[B21] Halford J. C. Gillespie J. Brown V. Pontin E. E. Dovey T. M. (2004). Effect of television advertisements for foods on food consumption in children. Appetite 42, 221–225. 10.1016/j.appet.2003.11.006 15010186

[B22] Hargrave S. L. Jones S. Davidson T. L. (2016). The outward spiral: a vicious cycle model of obesity and cognitive dysfunction. Curr. Opin. Behav. Sci. 9, 40–46. 10.1016/j.cobeha.2015.12.001 26998507 PMC4795925

[B23] Horstmann A. Dietrich A. Mathar D. Pössel M. Villringer A. Neumann J. (2015). Slave to habit? Obesity is associated with decreased behavioural sensitivity to reward devaluation. Appetite 87, 175–183. 10.1016/j.appet.2014.12.212 25543077

[B24] Kendig M. D. Boakes R. A. Rooney K. B. Corbit L. H. (2013). Chronic restricted access to 10% sucrose solution in adolescent and young adult rats impairs spatial memory and alters sensitivity to outcome devaluation. Physiol. Behav. 120, 164–172. 10.1016/j.physbeh.2013.08.012 23954407

[B25] Lachat C. Nago E. Verstraeten R. Roberfroid D. Van Camp J. Kolsteren P. (2012). Eating out of home and its association with dietary intake: a systematic review of the evidence. Obes. Rev. 13, 329–346. 10.1111/j.1467-789X.2011.00953.x 22106948

[B26] Mollen S. Rimal R. N. Ruiter R. A. Kok G. (2013). Healthy and unhealthy social norms and food selection. Findings from a field-experiment. Appetite 65, 83–89. 10.1016/j.appet.2013.01.020 23402712

[B27] Nelson A. Killcross S. (2006). Amphetamine exposure enhances habit formation. J. Neurosci. 26, 3805–3812. 10.1523/JNEUROSCI.4305-05.2006 16597734 PMC6674135

[B29] Ostlund S. B. Maidment N. T. Balleine B. W. (2010). Alcohol-paired contextual cues produce an immediate and selective loss of goal-directed action in rats. Front. Integr. Neurosci. 4:19. 10.3389/fnint.2010.00019 20725634 PMC2917216

[B30] Petrovich G. D. (2013). Forebrain networks and the control of feeding by environmental learned cues. Physiol. Behav. 121, 10–18. 10.1016/j.physbeh.2013.03.024 23562305 PMC3815748

[B31] Petrovich G. D. Ross C. A. Gallagher M. Holland P. C. (2007). Learned contextual cue potentiates eating in rats. Physiol. Behav. 90, 362–367. 10.1016/j.physbeh.2006.09.031 17078980 PMC1892280

[B32] Reppucci C. J. Petrovich G. D. (2012). Learned food-cue stimulates persistent feeding in sated rats. Appetite 59, 437–447. 10.1016/j.appet.2012.06.007 22721906 PMC3446245

[B33] Stroebele N. De Castro J. M. (2004). Effect of ambience on food intake and food choice. Nutrition 20, 821–838. 10.1016/j.nut.2004.05.012 15325695

[B34] Wansink B. (2004). Environmental factors that increase the food intake and consumption volume of unknowing consumers. Annu. Rev. Nutr. 24, 455–479. 10.1146/annurev.nutr.24.012003.132140 15189128

[B35] Weingarten H. P. (1983). Conditioned cues elicit feeding in sated rats: a role for learning in meal initiation. Science 220, 431–433. 10.1126/science.6836286 6836286

[B36] Willcocks A. L. McNally G. P. (2014). An extinction retrieval cue attenuates renewal but not reacquisition of alcohol seeking. Behav. Neurosci. 128, 83–91. 10.1037/a0035595 24512068

[B28] World Health Organisation (2016). Obesity and overweight. Fact sheet no. 311. Available online at: http://www.who.int/mediacentre/factsheets/fs311/en/

